# Self-reported health, function, and use of health care services in older prostate cancer survivors compared to matched controls: a cross-sectional study

**DOI:** 10.1007/s11764-024-01670-8

**Published:** 2024-09-17

**Authors:** Reidun Sletten, Marit Slaaen, Line Merethe Oldervoll, Håvard Kjesbu Skjellegrind, Jūratė Šaltytė Benth, Lennart Åstrøm, Øyvind Kirkevold, Sverre Bergh, Bjørn Henning Grønberg, Siri Rostoft, Asta Bye, Paul Jarle Mork, Ola Berger Christiansen

**Affiliations:** 1 Department of Public Health and Nursing, Faculty of Medicine and Health Sciences, Norwegian University of Science and Technology (NTNU), Trondheim, Norway; 2 The Research Center for Age-Related Functional Decline and Disease, Innlandet Hospital Trust, Ottestad, Norway; 3 Department of Oncology and Palliative Care, Innlandet Hospital Trust, Gjøvik/Lillehammer, Norway; 4 Institute of Clinical Medicine, Faculty of Medicine, University of Oslo, Oslo, Norway; 5 Centre for Crisis Psychology, Faculty of Psychology, University of Bergen, Bergen, Norway; 6 The National Institute on Intellectual Disability and Community, Department of Mental Health, Faculty of Medicine and Health Sciences, NTNU, Trondheim, Norway; 7 HUNT Research Centre, Department of Public Health and Nursing, Faculty of Medicine and Health Sciences, NTNU, Levanger, Norway; 8 Levanger Hospital, Nord-Trøndelag Hospital Trust, Levanger, Norway; 9 Institute of Clinical Medicine, Campus Ahus, University of Oslo, Oslo, Norway; 10 Health Services Research Unit, Akershus University Hospital, Lørenskog, Norway; 11 Section of Clinical and Experimental Oncology, Department of Immunology, Genetics and Pathology, Uppsala University, Uppsala, Sweden; 12 Faculty of Health, Care and Nursing, NTNU Gjøvik, Gjøvik, Norway; 13 The Norwegian National Centre for Ageing and Health, Vestfold Hospital Trust, Tønsberg, Norway; 14 Department of Clinical and Molecular Medicine, NTNU, Trondheim, Norway; 15 Department of Oncology, St.Olavs Hospital, Trondheim University Hospital, Trondheim, Norway; 16 Department of Urology, Innlandet Hospital Trust, Hamar, Norway; 17 Department of Geriatric Medicine, Oslo University Hospital, Oslo, Norway; 18 Department of Nursing and Health Promotion, Faculty of Health Sciences, Oslo Metropolitan University, Oslo, Norway

**Keywords:** Prostate cancer, Radical treatment, Cancer survivors, Self-reported health, Activities of daily living, Health care use

## Abstract

**Purpose:**

Information about outcomes of particular relevance to older prostate cancer survivors is limited. This study aimed to compare health, activities of daily living (ADL), and use of health care services between survivors and matched controls.

**Methods:**

A single-centre study on men treated for prostate cancer with curative intent at the age ≥ 70 years 2 to 7 years earlier. Controls matched on age and education were drawn (1:3) from the Trøndelag Health Study (HUNT) in Norway. Self-reported general health, independence in ADL and instrumental activities of daily living (IADL), hospital admissions and emergency room visits were compared by estimating non-adjusted and adjusted (age, education, comorbidity, cohabitant status and pack years of smoking) regression models.

**Results:**

The majority of both survivors (*N* = 233) and controls (*N* = 699) reported good (58.7% vs. 62.7%) or very good (11.2% vs. 6.8%) health and independence in ADL (95.6% vs. 96.3%) and IADL (82.7% vs. 81.9%). Hospital admission was reported by 17.3% vs. 18.2% and emergency room visit by 23.6% vs. 26.7%. Regression models showed no significant differences between survivors and controls.

**Conclusions:**

Older prostate cancer survivors reported similar health, independence in ADL and use of emergency room and hospital admissions as matched controls.

Implications for Cancer Survivors.

This study shows that survivors after curatively intended treatment of prostate cancer have as good health as matched controls, indicating that many patients tolerate such treatment well despite being of old age and that current practice for selection of patients offered such treatment is appropriate.

**Supplementary Information:**

The online version contains supplementary material available at 10.1007/s11764-024-01670-8.

## Introduction

With a global incidence of 1.5 million new cases in 2022, prostate cancer is the second most common cancer among men [1]. The median age at diagnosis is about 70 years, and in high-income countries, a major part is diagnosed with local or localized disease [2–4]. In this situation, curatively intended treatment, i.e. radical radiotherapy or surgery, may be offered, depending on tumour- and patient characteristics [5]. Older age seems to have been a major determinant for not offering radical treatment for prostate cancer [6–8]. It is, however, increasingly recognised that treatment of older adults should not be based on chronological age but rather on a systematic evaluation of health status and physiological and functional reserves [5, 9]. Following this, and a general improvement of health and longevity in older adults in an aging population, the number of older men undergoing curative treatment for prostate cancer is increasing [2, 10–12].

Higher age is associated with a gradual decline in functional and physical abilities, which may increase vulnerability to negative impacts of cancer and cancer treatment [13, 14]. More disabilities, poorer health and increased use of health services have been reported among older cancer survivors in comparison to older adults in general [15–18]. Whether this applies to older men after radical prostate cancer treatment is not known. The focus has mainly been on local side effects and urogenital functioning [19, 20]. Although highly prioritised outcomes [21] and an important basis for applying targeted supportive measures, few studies have investigated how older prostate cancer survivors perceive their health and ability to perform activities of daily living (ADL) [22–27]. Existing studies are either older [22, 27], lack comparison to age-matched controls [23–25] and/or include survivors irrespective of age [22, 24]. Moreover, many do not include follow-up beyond 12 months after treatment [25, 26], and the results are inconsistent [22, 24–26]. Available publications on health care use are also few, lacking separate analyses for older men [16, 22, 28].

In the present study, we aimed at assessing long-term general health, ADL, instrumental activities of daily living (IADL) and use of health care services in a cohort of older men having received radical treatment for prostate cancer, in comparison to a matched population-based control group. We also wanted to explore the impact of covariates like comorbidity and cohabitant status, known to be of major influence on older adults’ health status and utilisation of health services.

## Material and methods

### Study design and participants

In this cross-sectional single-centre study, we invited men who had received radical prostate cancer treatment at age 70 years or older between January 2014 and December 2018, and who were still alive in 2021. Treatment was either external beam radiotherapy (EBRT) or robotic-assisted radical prostatectomy (RARP). EBRT was combined with androgen deprivation therapy (ADT) for most patients. Further inclusion criteria were fluency in Norwegian and written informed consent. Eligible men were identified by the hospital’s electronic medical record (EMR). Invitation to participate, written information, the consent form and a self-report questionnaire were sent by postal mail. Those who consented to participate, returned a completed questionnaire and the signed consent form in two separate pre-paid envelopes. If no response was received within 4 weeks, one reminder was sent.

For comparison, a matched control group was drawn from the Trøndelag Health Study (The HUNT Study). HUNT is a population-based comprehensive health survey of the population in Trøndelag county, Norway [29, 30]. The study has been running in several waves since 1984, utilizing questionnaires, interviews, clinical examinations, laboratory measurements and collection of biological samples. The fourth wave (HUNT4) was conducted in 2017–2019 and included 56,042 (54% of invited) participants 20 years and older, who all answered a common first questionnaire (Q1). A second questionnaire (Q2) including age- and sex-specific questions representing part of a sub-study on older adults (HUNT4 70 +) was completed by approximately 10,000 participants ≥ 70 years [31]. A total of 2049 males were eligible as controls in the present study. Matching was performed on gender (all male), age at the time of inclusion (± 2 years) and on identical category of self-reported education. Three controls were selected for each prostate cancer survivor, using random selection among all possible matches.

### Assessments

The distributed questionnaires included items from Q1 and Q2 (male version) in HUNT4.

These covered sociodemographic measures like cohabitant status and educational attainment, health-related information including general health, smoking history, comorbidity, ADL, IADL and use of health care services.

Cohabitant status was dichotomized from self-report of either living alone or living with other persons (partner/spouse, other adults or children). Educational attainment was defined as the highest self-reported level of education on a four-category variable (primary school, high school, vocational education or college/university). For registration of comorbidity, the survivors answered “yes” or “no” to ever having had 18 defined medical conditions, giving a summary score ranging from 0 to 18. The condition “cancer” in the original HUNT4 questionnaire was changed to “cancer other than prostate cancer”. Smoking pack years were calculated from smoking habits and age. One pack year was defined as smoking 20 cigarettes a day for 1 year and defined as 0 if the response was “I have never smoked”. Information about the use of health care services was collected by asking if such services (e.g. visits to the general practitioner (GP), hospital admissions, emergency room visits, and home care nursing) had been used during the last 12 months (yes/no). Information on the survivors’ prostate cancer, including treatment and clinical relapse, was obtained from their EMR. Clinical relapse was defined as the occurrence of distant metastasis or additional treatment (salvage radiotherapy or surgery, lifelong androgen deprivation therapy, chemotherapy, or other medical treatment for recurrent disease).

### *Outcome measures*

The primary outcome was self-perceived general health from Q1, HUNT4, measured by the single question “What is your current health status?” answered on a 4-point Likert scale from “poor” to “very good”. The question captures the subjective perception of health and has been shown to predict mortality [32, 33].

Secondary outcomes were independence in ADL and IADL, and hospital admissions and emergency room visits, all defined by items from HUNT4. ADL covered basic personal tasks, i.e. moving around indoors on the same floor, washing, bathing/showering, dressing, toileting, eating and getting in and out of bed. IADL covered more advanced daily tasks, i.e. preparing warm meals, light and heavy housework, doing laundry and shopping, paying bills, taking medication, going out and taking the bus [34, 35]. Participants were classified as independent in each domain if able to do all included tasks without assistance. Information about hospital admissions and emergency room visits was obtained from the question on the use of health care services, where the answer “yes” meant the specified health care had been used during the last 12 months.

### Ethics

The project was approved by the Regional Committee for Medical Research Ethics Southeast Norway (REK South-East) (ID 183868, on 10th of March 2021) and Innlandet Hospital Trust’s official data protection officer and registered in ClinicalTrials.gov (NCT04863352, on 27th of April 2021). The procedures used in this study adhere to the tenets of the Declaration of Helsinki. All participants provided written informed consent.

### Statistical analysis

Descriptive statistics is reported by means and standard deviations (SDs) for continuous variables and as frequencies and percentages for categorical variables. Since within-pair correlations might be present due to matching, the characteristics of the prostate cancer survivors and the matched controls were compared by generalized linear mixed models. In the case of convergence problems, independent-samples *t*-test, *χ*^2^-test or Fisher’s exact test was applied.

Self-perceived general health, the four-category primary outcome, was compared between the cohort of survivors and the matched control group by estimating a multinomial logistic regression model with “good” as reference category. Ordinal regression was unsuitable due to violation of the proportional odds assumption. The results were presented as odds ratios (ORs) and corresponding 95% confidence intervals (CIs) quantifying the difference between survivors and controls in odds for “poor”, “not so good” or “very good” vs. the reference (“good”). The model was adjusted for factors that reportedly have an impact on health and function, i.e. social status (cohabitant status), comorbidities and smoking history (pack years) [36–38]. Age and education are also important [14, 39], and although matched for, we adjusted for age to correct for potential residual confounding. A corresponding correction for educational attainment was not possible due to small sample group sizes.

Logistic regression models were estimated to address the secondary outcomes ADL and IADL, dichotomized into “independent” (able to do all ADL/IADL tasks without assistance) or “dependent” (needing help with one or more ADL/IADL task), hospital admissions, dichotomized into having been admitted to hospital last 12 months (yes/no), and likewise emergency room visits dichotomized into having visited the emergency room last 12 months (yes/no). Adjustments were made for the same factors as in the primary outcome analyses. In addition, educational attainment was also considered, as groups were large enough for these analyses.

No cluster effect within pairs of survivors and controls was found, thus no adjustment was needed in the above analyses.

The proportion of missing values among covariates was high in the regression analyses (nearly 30% of cases excluded). Excluded and included cases were therefore compared by independent-samples *t*-test or *χ*^2^-test. To minimize potential bias due to high proportion of missing, the normalized inverse probability weights were generated and used in regression analyses. Normalization of weights was performed so that the pseudo-sample generated by weighting is of the same size and thus standard errors are not inflated by artificially increased sample size.

To avoid uncertainties related to any cancer relapse, sensitivity analysis including only men without relapse was performed for both primary and secondary outcomes.

## Results

### Study cohort and characteristics

Overall, 233 of 345 (67.5%) eligible prostate cancer survivors participated in the study, the remaining 112 (32.5%) either declined or did not answer. Of those participating, 126 (54.1%) had received RARP, 107 (45.9%) had received EBRT and the mean time since treatment was 55.4 months (Table 1). Clinical relapse was registered for 42 (18.0%). For comparison, 699 matched controls were drawn from HUNT 4. Demographic characteristics were similar in the two groups (Table 1). Mean (SD) age was 78.4 (3.1) years, 33.2% of survivors vs. 33.3% of controls had university or college education and the majority (79.3% vs. 78.0%) was living with someone. The mean number of comorbidities was 1.5 (SD 1.6) in survivors vs. 1.6 (1.5) in controls.

**Table 1:** Characteristics of the prostate cancer survivors and comparison to matched controls

Covariate	Survivors *N* = 233	Matched controls *N* = 699	*p*-value
Age^a^, mean (SD)	78.4 (3.1)	78.4 (3.1)	0.41^b^
Educational attainment^a^, *n* (%)			
Primary school	43 (19.0)	132 (18.9)	1.00^c^
High school	57 (25.2)	178 (25.5)	
Vocational education	51 (22.6)	156 (22.3)	
College/university	75 (33.2)	233 (33.3)	
*n*	226	699	
Cohabitant status, *n* (%)			
Living alone	47 (20.7)	153 (22.0)	0.68^d^
Living with others (included partner/spouse)	180 (79.3)	542 (78.0)	
*n*	227	695	
Number of comorbidities, *n* (%)			
0–1 comorbidity	112 (58.6)	320 (55.8)	
2–5 comorbidities	77 (40.3)	242 (42.2)	
> 5 comorbidities	2 (1.0)	11 (1.9)	
Mean (SD)	1.5 (1.6)	1.6 (1.5)	0.47^b^
*n*	191	573	
Smoking, pack years			
0 years	108 (50.9)	264 (43.3)	
< 1–5 years	15 (6.4)	59 (9.7)	
6–10 years	16 (6.9)	58 (9.5)	
> 10 years	73 (31.3)	229 (37.5)	
Mean (SD)	11.3 (18.4)	11.6 (17.4)	0.83^b^
*n*	212	610	
Treatment and disease status in survivors, *n* (%)			
RARP^e^	126 (54.1)	-	-
EBRT^f^	107 (45.9)		
Adjuvant ADT^g^	99 (92.5^ h^)	-	-
No adjuvant ADT	8 (7.5^ h^)		
Recurrent disease/relapse	42 (18)	-	-
Life-long ADT for recurrent disease, mean (SD)	24 (10.3)		
Months after treatment, mean (SD)	55.4 (17.6)	-	-
*n*	233		

^a^Matching variables; ^b^linear mixed model; ^c^*χ*^2^-test (generalized linear mixed model did not converge); ^d^generalized linear mixed model; ^e^*RARP*, robotic-assisted radical prostatectomy; ^f^*EBRT*, external beam radiotherapy; ^g^*ADT*, androgen deprivation therapy; ^h^percent of primary treatment EBRT (total *n* = 107)

### General health, ADL and IADL

Missing answers for these primary and secondary outcomes varied between groups. For survivors vs. controls, the missing proportion for general health was 4.3% vs. 2.6%, for ADL 6.4% vs. 10.2% and for IADL 6.0% vs. 15.3%.

Most survivors and controls reported their general health as good (58.7% vs. 62.7%) or very good (11.2% vs. 6.8%) (Table 2). Not-so-good health was reported by 26.5% vs. 29.2% and poor health by 3.6% vs. 1.3%. Independency in ADL was reported by 95.6% of the survivors vs. 96.3% of the controls, whereas 4.4% vs. 3.7% had dependency in one or more activities (Table 2, Supplementary Table 1). For IADL, 82.6% of the survivors and 81.9% of controls reported being independent (Table 2). Any dependency was reported by 17.4% and 18.1%, respectively, the majority having only one. The most frequent dependencies in both groups were related to laundry and heavier housework (Supplementary Table 2).

**Table 2:** General health, daily life activities and use of health care services in prostate cancer survivors in comparison to matched controls from a normal population of the same gender, age and education

	Survivors *N* = 233	Matched controls *N* = 699
General health and daily life activities, *n* (%)		
Self-rated general health		
Poor	8 (3.6)	9 (1.3)
Not so good	59 (26.5)	199 (29.2)
Good	131 (58.7)	427 (62.7)
Very good	25 (11.2)	46 (6.8)
*n*	223	681
Activities of daily living (ADL)		
Dependent in one or more	10 (4.4)	23 (3.7)
Independent	218 (95.6)	605 (96.3)
*n*	228	628
Instrumental activities of daily living (IADL)		
Dependent in one or more	38 (17.4)	107 (18.1)
Independent	181 (82.6)	485 (81.9)
*n*	219	592
Use of health care services last 12 months, *n* (%)		
Emergency room visit		
No	159 (76.4)	469 (73.3)
Yes	49 (23.6)	171 (26.7)
*n*	208	640
Hospital admission		
No	187 (82.7)	560 (81.8)
Yes	39 (17.3)	125 (18.2)
*n*	226	685
Hospital, somatic out-patient visit		
No	156 (74.3)	441(67.6)
Yes	54 (25.7)	211 (32.4)
*n*	210	652
Visit to specialist outside hospital		
No	172 (86.0)	530 (86.3)
Yes	28 (14.0)	84 (13.7)
*n*	200	614
Visit to general practitioner (GP)		
No	8 (3.5)	41 (6.0)
Yes	220 (96.5)	648 (94.0)
*n*	228	689
Nursing home admission		
No	220 (95.2)	605 (96.0)
Yes	11 (4.8)	25 (4.0)
*n*	231	630
Received home care nursing		
No	225 (97.4)	617 (97.8)
Yes	6 (2.6)	14 (2.2)
*n*	231	631

According to the adjusted multinomial logistic regression model with normalized inverse probability weights, the odds for reporting health as “poor” rather than “good” were higher for survivors than for controls (adjusted OR 12.25, 95% CI 2.35; 63.94, Table 3). Otherwise, there were no statistically significant differences in odds between the survivors and controls, neither related to general health nor independence in ADL or IADL (Table 4).

**Table 3:** Results from nominal regression models for self-perceived general health in prostate cancer survivors in comparison to matched controls from a normal population of the same gender, age and education

Covariate	Unadjusted models		Adjusted models	
	OR^a^ (95% CI^b^)	p-value	OR^a^ (95% CI^b^)	p-value
Pair, survivor (control – ref.)				
Poor	9.54 (1.89; 48.08)	0.006*	12.25 (2.35; 63.94)	0.003*
Not so good	0.78 (0.50; 1.22)	0.281	0.83 (0.52; 1.33)	0.442
Good – ref.	1	-	1	-
Very good	1.74 (0.96; 3.15)	0.066	1.50 (0.81; 2.78)	0.198
Age				
Poor	1.06 (0.87; 1.30)	0.559	1.08 (0.85; 1.36)	0.523
Not so good	1.00 (0.95; 1.06)	0.919	0.98 (0.92; 1.05)	0.544
Good – ref.	1	-	1	-
Very good	0.98 (0.88; 1.08)	0.623	0.98 (0.89; 1.08)	0.675
Cohabitant status, Living alone (Living with others – ref)				
Poor	0.58 (0.07; 4.81)	0.617	0.48 (0.13; 1.77)	0.269
Not so good	1.46 (0.95; 2.22)	0.081	1.56 (1.00; 2.43)	0.049*
Good – ref.	1	-	1	-
Very good	0.59 (0.26; 1.36)	0.216	0.55 (0.22; 1.35)	0.190
Comorbidities				
Poor	2.31 (1.65; 3.23)	<0.001*	2.57 (1.52; 4.33)	<0.001*
Not so good	1.53 (1.35; 1.74)	<0.001*	1.53 (1.35; 1.75)	<0.001*
Good – ref.	1	-	1	-
Very good	0.52 (0.37; 0.72)	<0.001*	0.53 (0.36; 0.76)	0.001*
Smoking, pack years				
Poor	1.04 (1.00; 1.06)	0.007*	1.04 (1.01; 1.07)	0.013*
Not so good	1.00 (0.99; 1.01)	0.548	1.00 (0.99; 1.01)	0.736
Good – ref.	1	-	1	
Very good	0.96 (0.92; 0.99)	0.012*	0.96 (0.93; 0.996)	0.030*

*Significance level *p* < 0.05

^a^*OR* odds ratio, ^b^*CI* confidence interval

**Table 4:** Results from regression models for the secondary outcomes: independence in activities of daily living (ADL) and instrumental ADL (IADL), and admission to hospital and emergency room visits the last 12 months in prostate cancer survivors compared to matched controls from a normal population of the same gender, age and education

	**Independence in activities of daily living (ADL)** ^**a**^ ***n*** **=623 (**normalized IPW used);				**Independence in instrumental ADL (IADL)** ^**b**^ ***n*** **=597** (normalized IPW used)			
	Unadjusted model		Adjusted model		Unadjusted model		Adjusted model	
Covariate	OR^**c**^ (95% CI ^**d**^)	p-value	OR (95% CI)	p-value	OR (95% CI)	p-value	OR (95% CI)	p-value
**Survivor** (vs. control – ref.)	1.02 (0.37; 2.80)	0.972	1.05 (0.37; 3.01)	0.922	0.84 (0.53; 1.35)	0.480	0.89 (0.53; 1.49)	0.662
**Age**	0.94 (0.83; 1.06)	0.297	0.92 (0.82; 1.04)	0.170	0.88 (0.82; 0.94)	< 0.001*	0.88 (0.82; 0.95)	< 0.001*
**Educational attainment**								
Primary school	0.35 (0.09; 1.37)	0.133	0.41 (0.11; 1.47)	0.171	0.44 (0.24; 0.82)	0.010*	0.47 (0.25; 0.90)	0.021*
High school	0.75 (0.21; 2.76)	0.669	0.88 (0.25; 3.10)	0.841	0.43 (0.24; 0.77)	0.005*	0.47 (0.26; 0.85)	0.012*
Vocational education	0.70 (0.18; 2.64)	0.596	0.93 (0.24; 3.68)	0.920	1.10 (0.57; 2.15)	0.776	1.35 (0.68; 2.70)	0.394
College/university – ref.	1	-	1	-	1	-	1	-
**Cohabitant status, Living alone**								
(Living with others – ref)	1.39 (0.42; 4.61)	0.586	1.96 (0.56; 6.81)	0.289	1.19 (0.66; 2.15)	0.571	1.59 (0.86; 2.91)	0.137
**Comorbidities**	1.04 (0.77; 1.41)	0.779	1.10 (0.78; 1.54)	0.582	0.83 (0.73; 0.95)	0.006*	0.82 (0.71; 0.95)	0.007*
**Smoking, pack years**	0.98 (0.96; 0.998)	0.031*	0.97 (0.95; 0.99)	0.016*	0.98 (0.97; 0.99)	0.004*	0.98 (0.97; 0.99)	0.002*
	**Hospital admission** ^**e**^ ***n*** **=661** (normalized IPW used)				**Emergency room visit** ^**f**^ ***n*** **=622** (normalized IPW used)			
	Unadjusted model		Adjusted model		Unadjusted model		Adjusted model	
Covariate	OR (95% CI)	p-value	OR (95% CI)	p-value	OR (95% CI)	p-value	OR (95% CI)	p-value
**Survivor** (vs. control – ref.)	1.09 (0.69; 1.74)	0.703	1.15 (0.72; 1.82)	0.567	0.88 (0.58; 1.35)	0.567	0.90 (0.58; 1.39)	0.622
**Age**	0.99 (0.93; 1.06)	0.879	0.97 (0.91; 1.04)	0.383	1.02 (0.95; 1.09)	0.533	1.00 (0.93; 1.08)	0.931
**Educational attainment**								
Primary school	1.22 (0.61; 2.43)	0.581	1.25 (0.61; 2.53)	0.541	1.23 (0.71; 2.13)	0.466	1.22 (0.70; 2.11)	0.487
High school	1.23 (0.71; 2.12)	0.462	1.33 (0.76; 2.31)	0.313	1.49 (0.88; 2.51)	0.135	1.55 (0.91; 2.64)	0.108
Vocational education	1.49 (0.85; 2.60)	0.161	1.55 (0.87; 2.76)	0.137	1.69 (0.98; 2.89)	0.057	1.71 (1.00; 2.93)	0.051
College/university – ref.	1	-	1	-	1	-	1	-
**Cohabitant status, Living alone**								
(Living with others – ref)	1.48 (0.91; 2.41)	0.117	1.58 (0.96; 2.59)	0.069	1.26 (0.82; 1.93)	0.285	1.30 (0.84; 2.00)	0.242
**Comorbidities**	1.27 (1.13; 1.43)	<0.001*	1.29 (1.14; 1.46)	<0.001*	1.20 (1.07; 1.35)	0.001*	1.21 (1.08; 1.36)	0.001*
**Smoking, pack years**	1.00 (0.99; 1.01)	0.691	0.99 (0.98; 1.00)	0.220	1.00 (0.99; 1.01)	0.756	0.99 (0.98; 1.01)	0.304

*Significance level *p* < 0.05

^**a**^Logistic regression model for independence in ADL, OR for value 1 (“Independent”); ^**b**^ Logistic regression model for independence in IADL (OR for value 1 (“Independent”), ^**c**^ OR – odds ratio; ^**d**^CI – confidence interval; ^**e**^ logistic regression model (adjusted for intra-pair correlations) for hospital admission, (OR for value 1 (“Yes”); ^e^Logistic regression model adjusted for intra-pair correlations for emergency room visit (OR for value 1 (“Yes”)

### Use of health care services

Missing answers on health care use varied between items but were comparable between groups, except for nursing home admission and use of home care. On these items, information was missing from 0.8% of the cancer survivors and 9.7–9.9% of the controls.

Overall, 23.6% of the prostate cancer survivors vs. 26.7% of the controls reported one or more emergency room visits during the last 12 months, 17.3% vs. 18.2% had been admitted to hospital, and the majority in both groups (i.e. 96.5% vs. 94.0%) had visited their GP (Table 2). Hospital out-patient visits and consultations with a specialist outside the hospital were less frequent, and very few had been admitted to a nursing home (4.8% of survivors vs. 4.0% of controls) or had received home care nursing (2.6% vs. 2.2%) (Table 2). The use of health care services was thus comparable between survivors and controls. For the secondary outcomes, this was confirmed by the logistic regression models, showing no significant differences between the prostate cancer survivors and the matched control group in hospital admission and emergency room visits (Table 4).

### Association between outcomes, comorbidity and other confounders

Several of the factors adjusted for in the adjusted regression analyses had a significant association with one or more of the pre-defined outcomes (Tables 3 and 4). A higher number of comorbidities was associated with higher odds for reporting “poor” rather than “good” (OR 2.57 [1.52; 4.33]), or “not so good” rather than “good” general health (OR 1.53 [1.35; 1.75]), having emergency room visits (OR 1.21 [1.08; 1.36]) or being admitted to hospital (OR 1.29 [1.14; 1.46]). Comorbidities were also associated with lower odds for reporting “very good” rather than “good” health (OR 0.53 [0.36; 0.76]) and being independent in IADL (OR 0.82 [0.71; 0.95]). Number of smoking pack years was associated with higher odds for reporting “poor” rather than “good” health (OR 1.04 [1.01; 1.07]), and lower odds for reporting health as “very good” rather than “good” (OR 0.96 [0.92; 0.99]), and being independent in ADL (OR 0.97 [0.95; 0.99]) and IADL (OR 0.98 [0.97; 0.99]). Moreover, educational attainment and age were associated with independence in IADL, and living alone was associated with higher odds for “not so good” rather than “good” self-reported health.

Sensitivity analyses including only prostate cancer survivors without clinical relapse, showed comparable results to the main analysis for all outcomes, though a separate analysis on the group who had “poor” self-reported health could not be run due to small group sizes (data not shown).

## Discussion

In this study on older prostate cancer survivors, who received radical treatment 2 to 7 years earlier, we found that the majority reported their general health as good and was as independent in ADL and IADL as a group of matched controls from a general population. There was also no difference between the survivors and the control group in hospital admissions and emergency room visits.

Our study cohort had a mean age of 78.4 years, reflecting a time in life where functional and physical abilities may gradually deteriorate for several reasons, e.g. aging itself, comorbidities or frailty development. The deterioration may be aggravated by cancer and cancer treatment [13–15, 18]. An older Norwegian study opposed our findings by showing worse self-reported health in prostate cancer survivors (all ages) compared to population-based controls [22]. These results may however be less relevant, as treatment and treatment strategies have significantly changed in the three decades since the survivors in that study were diagnosed with prostate cancer [11]. There was no information on whether survivors in that study had received any radical treatment for their cancer [22]. In accordance with our results, two previous studies reported comparable functioning between a non-cancer control group and older (≥ 65 years) survivors about 12 months after treatment for localised prostate cancer [26, 27]. A broad UK survey also supports our findings, showing that perceived health in prostate cancer survivors did not diverge from mean population-based scores [24]. In contrast to two previous studies [16, 28], we did not find any increase in the use of health care services in prostate cancer survivors. The reason may not only be differences in population characteristics [16, 28] but also that our data did not include sufficient details (e.g. number of hospital admissions or emergency room visits) to uncover any distinction between groups. The result does, however, fit with the comparability of survivors and controls on our other outcomes.

Overall, our study extends the knowledge on prostate cancer survivors’ perceived health and daily life functioning beyond the previously reported 12 months in studies including non-cancer controls [26, 27]. Further, it suggests that in the longer run, radical treatment was well tolerated. This is reassuring but may also indicate that treatment was offered in accordance with health status [5, 9]. A low number of comorbidities in our survivors supports this assumption. Moreover, the negative impact of comorbidities and smoking pack years is in line with established knowledge [25, 36, 38, 40] and emphasizes the importance of measuring such indicators of vulnerability.

Our findings indicate that the long-term needs of older prostate cancer survivors related to general health and ADL do not differ from the general older male population. It must be kept in mind that these are long-term results for survivors and refer to a time several years after the end of treatment, where acute treatment side effects have subsided. Despite comparable results between survivors and controls in this setting, survivors may have increased needs for rehabilitation in the shorter term after curative treatment, as indicated by two previous studies [25, 27].

## Strengths and limitations

The study has several strengths. The main one is the comparison with matched controls from a general population. Furthermore, the sample size is relatively large, the response rate is high and the assessments are made by validated instruments.

However, several limitations must be considered. A major one is the cross-sectional design and lack of baseline assessments. This means that although current findings are comparable, the survivors may have experienced a more pronounced decline or may have been worse off earlier in the disease trajectory (25, 27). Second, although other factors, in particular comorbidity, are strongly associated with our outcomes, due to the cross-sectional design, we do not know whether they were influenced by the previous cancer diagnosis or treatment. Possibly, comorbidity could have been worsened because of cancer treatment. Third, the time frame between treatment and assessment varied largely. For all men, however, the period was no shorter than 2 years, meaning that the acute phase of side effects was well passed, and their situation stabilized. Fourth, missing responses reduced sample sizes in the regression models, and thus potentially made the results of the models less robust. We addressed this through normalized inverse probability weights to minimize bias. Finally, our population was heterogeneous in terms of treatment modality. Generally, patients accepted for surgery are healthier than patients accepted for radiotherapy. Separate analysis would have been ideal, but to retain statistical power in the regression analyses, we chose joint analysis. This strategy made it possible to adjust for covariates such as comorbidity, smoking pack years and cohabitant status, which we presumed and documented to be of major importance for most outcomes.

## Conclusions

In summary, this study adds important knowledge on what to expect for older men with prostate cancer in the long run. We found that 2–7 years after radical treatment, most older men were doing well, lived independent lives, and reported a pattern of health care use similar to matched controls. Comorbidity and smoking history seemed to be important for long-term general health and function, underlining the importance of assessing such factors in survivorship care.

## Supplementary Information

Below is the link to the electronic supplementary material.

Supplementary file1 (DOCX 13 KB)

## Data Availability

Due to a statement by the Data Protection Officer at Innlandet Hospital Trust, and in accordance with Norwegian privacy regulations, data cannot be shared publicly because they are confidential (due to the consent given by the men when included in the study). It is possible to extract information, upon request. Proposals should be directed to the Research Department of Innlandet Hospital Trust; contact: SIHFDLforskning@sikt.sykehuspartner.no.
