# Supplementary material for: Self-reported health, function, and use of health care services in older prostate cancer survivors compared to matched controls: a cross-sectional study
Source: J Cancer Surviv. 2024 Sep 17;20(2):543–53. doi: 10.1007/s11764-024-01670-8 (PMC12989016; doi:10.1007/s11764-024-01670-8)
Supplement: Supplementary file 1 — Supplementary file1 (DOCX 13 KB) [file 11764_2024_1670_MOESM1_ESM.docx]

## Supplementary tables

**Supplementary table 1**. Items with dependency in activities of daily living (ADL) in prostate cancer survivors in comparison to matched controls from a normal population of the same gender, age and education.

|  | **Survivors (n = 233)** | **Controls (n = 699)** |
| --- | --- | --- |
| Item with registered dependency | n (%) | n (%) |
| Move around indoors on the same floor | 8 (3) | 17 (2) |
| Go to the toilet | 8 (3) | 17 (2) |
| Wash yourself | 9 (4) | 19 (3) |
| Take a bath or shower | 10 (4) | 24 (3) |
| Dress and undress yourself | 9 (4) | 18 (3) |
| Go to bed and get up | 8 (3) | 18 (3) |
| Eating | 8 (3) | 15 (2) |

Mean number of missing items 4.1 (1.8%) for survivors and 66 (9.4%) for controls

**Supplementary table 2**. Items with dependency in instrumental activities of daily living (IADL) in prostate cancer survivors in comparison to matched controls from a normal population of the same gender, age and education.

|  | **Survivors (n = 233)** | **Controls (n = 699)** |
| --- | --- | --- |
| Item with registered dependency | n (%) | n (%) |
| Prepare warm meals | 7 (3) | 25 (4) |
| Do light housework (e.g., wash dishes) | 5 (2) | 14 (2) |
| Do heavier housework (e.g., wash floor) | 22 (9) | 53 (8) |
| Do the laundry | 19 (8) | 69 (10) |
| Do the shopping | 9 (4) | 16 (2) |
| Pay bills | 16 (7) | 37 (5) |
| Take the medicines | 4 (2) | 10 (1) |
| Go out | 3 (1) | 5 (1) |
| Take the bus | 17 (7) | 20 (3) |

Mean number of missing items 5.6 (2.4%) for survivors and 74.2 (10.6 %) for controls
